# Immunoglobulin a (IgA) Vasculitis in the Elderly

**DOI:** 10.7759/cureus.34422

**Published:** 2023-01-31

**Authors:** Adriana Girao, José A Fernandes, Filipe S Mira, Rui Pina

**Affiliations:** 1 Internal Medicine Department, Centro Hospitalar e Universitário de Coimbra, Coimbra, PRT; 2 Nephrology Department, Centro Hospitalar e Universitário de Coimbra, Coimbra, PRT

**Keywords:** elderly population, glomerular iga staining, iga nephropathty, ig a vasculitis, henoch-schönlein purpura (iga vasculitis), palpable purpura

## Abstract

IgA vasculitis is a small vessel vasculitis mediated by the deposition of IgA immune complexes. It mostly occurs in children and is rare in adults, with increased severity and mortality in the latter. Its aetiology remains largely unknown, and its prognosis depends primarily on the extent of renal involvement. We present the case of a 71-year-old woman with purpuric lesions in both lower and upper limbs associated with fever, abdominal pain, vomiting and blood in her stools for the past month. The patient was diagnosed with IgA vasculitis and the full systemic involvement (renal, dermatological, intestinal, and cerebral) of the disease was identified with excellent response to parenteral corticotherapy.

## Introduction

Immunoglobulin A vasculitis (IgAV), previously named Henoch-Schonlein Purpura (HSP), is the most common systemic vasculitis of childhood and is two to 33 times more common in children than in adults. The estimated annual incidence is 0.8-1.8/100,000 for adults [1]. Most studies show a male-to-female ratio of 1.2:1 to 1.8:1. Rarely occurring in summer months, IgAV primarily occurs in the fall, winter, and spring, which can be possibly explained by the association of IgAV with infections. Though the underlying cause of IgAV remains unknown, it is thought that it represents an immune-mediated small vessel vasculitis that could be triggered by a variety of antigens [2]. About half of the cases of IgAV are preceded by an upper respiratory tract infection, primarily in children. Other triggers have been studied such as infectious agents, vaccination, and insect bites. The most consensual diagnostic criteria were developed by the European Alliance of Association for Rheumatology (EULAR) and the Paediatric Rheumatology European Society (PRES), being posteriorly validated by the Paediatric Rheumatology International Trials Organization (PRINTO). The mandatory criterion for diagnosis is the presence of purpura or petechiae, most predominant in the lower limbs, without coagulopathy or thrombocytopenia. One or more criteria are also necessary - kidney involvement, such as hematuria or proteinuria, abdominal pain, usually diffuse and of acute onset, leukocytoclastic vascultitis or proliferative glomerulonephritis, with predominant IgA deposits, and arthritis or arthralgia (acute onset) [3].

## Case presentation

**Figure 1:**
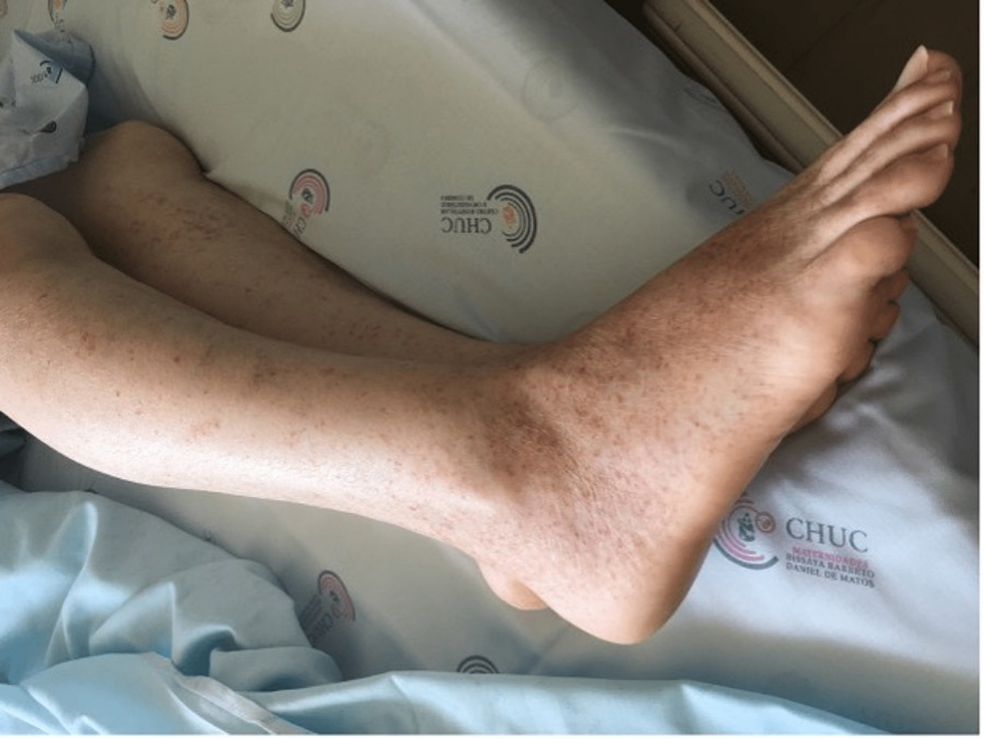
Palpable purpuric lesions on the legs

**Figure 2:**
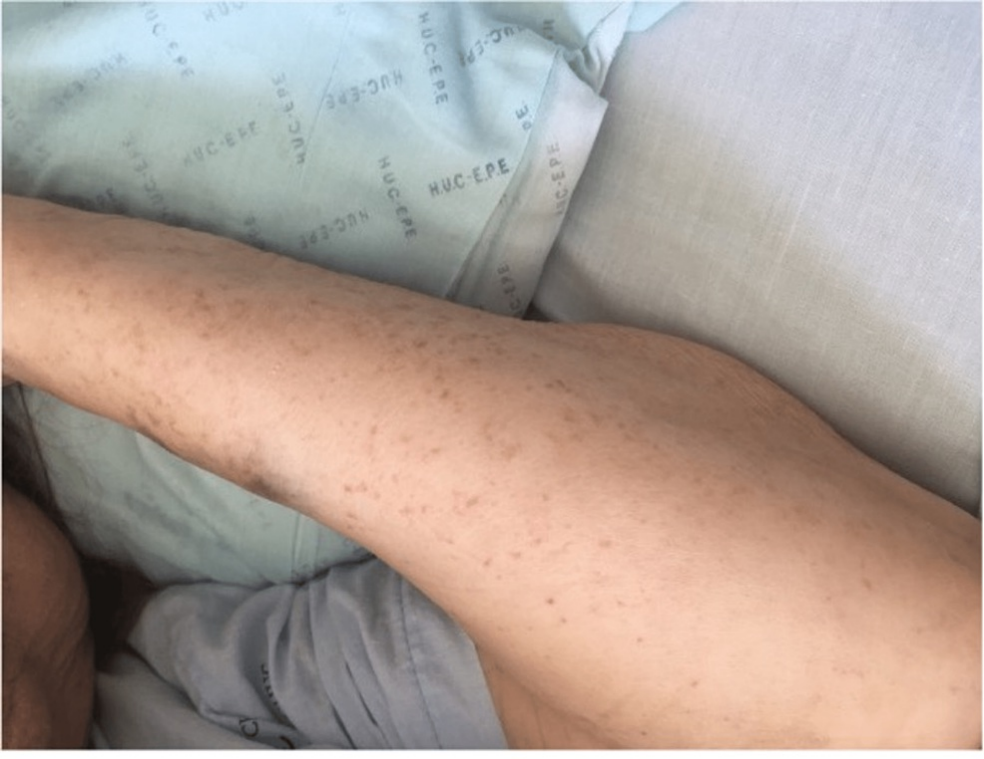
Palpable purpuric lesions on the arms

A 71-year-old Caucasian woman presented to the Emergency Department (ED) with a month-old polyarthralgia/myalgia and palpable purpuric lesions on the lower and upper limbs (Figures 1, 2).

She had a fever (38ºC) with abdominal pain and nausea, with bouts of vomiting and bloody diarrhea. The urinalysis revealed proteinuria and hemoglobinuria before catheterization and the kidney/bladder ultrasound showed signs of functional compromise (reduced kidney dimensions and loss of cortico-medullary differentiation). Routine bloodwork showed high C reactive protein of 5.91mg/dL with leukocytosis of 16x10^9^/L. Serum creatinine (SCr) was normal (0.80mg/dL) with an estimated Glomerular function ratio (eGFR) of 79mL/min/1.73m^2^.

**Figure 3:**
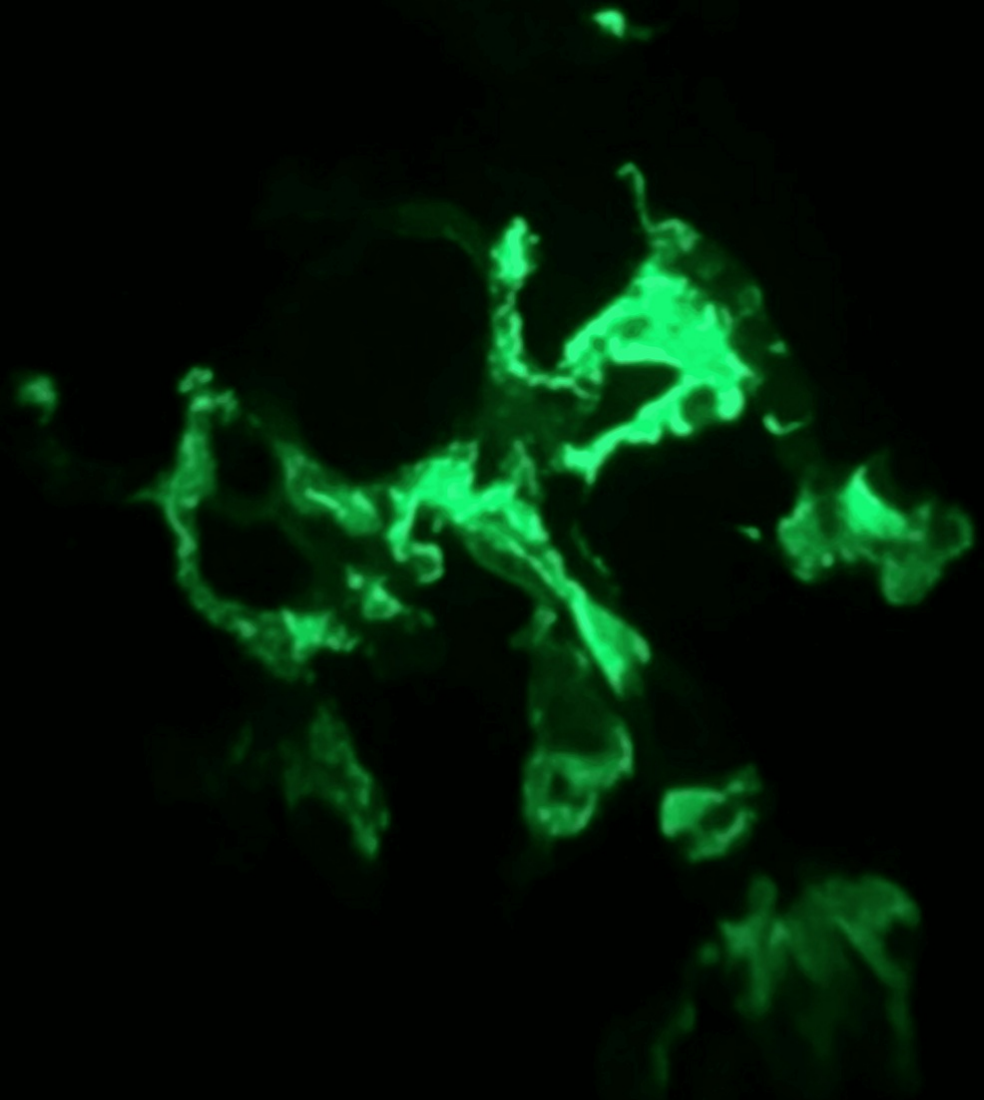
Glomerular IgA staining by immunofluorescence microscopy

She was admitted to the Internal Medicine ward for clinical stabilization and etiologic study. She was tested for infectious diseases (Epstein Barr virus, Cytomegalovirus, Human Immunodeficiency virus, Hepatitis B and C virus, Toxoplasmosis, Syphilis, Parvovirus and Herpes Simplex virus 1/2), autoimmune diseases, blood, urine and stool cultures. The exams showed subnephrotic proteinuria on a 24h collection (2252mg/dL) and worsening of kidney function with SCr that rose to 1.19mg/dL with an eGFR of 49mL/min/1.73m^2^. The next step was a kidney biopsy which showed the presence of IgA deposits in immunofluorescence staining, compatible with IgA nephropathy (Figure 3).

Due to the bloody stools and abdominal pain an endoscopic study was performed, which revealed multiple ulcerations on segment II of duodenus, compatible with peptic duodenitis; colonoscopy was normal. A brain computer tomography scan (CT) was also performed due to signs of early dementia with abnormal behaviour such as social disinhibition, which showed lesions compatible with microangiopathic insult. Due to the absence of improvement of the skin lesions with topic corticotherapy, a skin biopsy was also performed confirming the presence of IgA deposits.

The diagnosis of IgA vasculitis was assumed, and the patient was medicated with prednisolone 1mg/kg/day, with significant improvement of kidney function and urinary sediment, as well as skin lesions and behavioural issues. After a 38-day admission, the patient was discharged with an SCr of 0.79mL/dL, returning to pre-admission values. She was discharged with Internal Medicine and Nephrology follow-up, with great response to therapy after six months and one year.

## Discussion

By the EULAR/PRINTO/PRESS criteria, the patient presented all criteria: purpuric lesions (mandatory criteria); abdominal pain with gastrointestinal bleeding; histopathologic documentation of IgA deposits; arthralgia and renal involvement with proteinuria and hematuria [3]. Having all the criteria is an unusual presentation, with central nervous system involvement being rare but more common in adults and patients with severe forms of the disease [4]. Adults frequently have a worse prognosis, often associated with renal involvement. Age over 65 years, nephrotic proteinuria, acute kidney injury, and hematuria are usually associated with the worst prognosis [5,6]. Systemic corticotherapy should be considered for stabilization of the disease, improving not only quality of life but also disease prognosis. Early corticosteroid treatment significantly reduced the odds of developing the persistent renal disease [7]. However, the complications of steroid therapy, especially in the elderly, must the taken into consideration such as increased susceptibility to infection and femoral neck fractures [8]. Impaired renal function in the elderly could result in higher blood concentrations of steroids, and prolonged therapy could result in immune suppression [9]. Steroid therapy should be short and in the lowest dose needed to reduce the possibility of side effects.

## Conclusions

IgAV in adults, particularly in the elderly, is an uncommon and underdiagnosed condition. This case report, with all the EULAR/PRINTO/PRESS criteria, is a rare case, with most cases only presenting two or three criteria. Even though this case presented with clinical features associated with a poorer prognosis, clinical suspicion and the early corticotherapy treatment were the key to the overall success. In conclusion, this report highlights that when examining elderly patients with palpable purpura and abdominal pain, the possibility of IgAV should be considered.
